# Genome-Wide Analysis of Carboxylesterases (COEs) in the Whitefly, *Bemisia tabaci* (Gennadius)

**DOI:** 10.3390/ijms20204973

**Published:** 2019-10-09

**Authors:** Jixing Xia, Haifeng Xu, Zezhong Yang, Huipeng Pan, Xin Yang, Zhaojiang Guo, Fengshan Yang, Litao Guo, Xiaodong Sun, Shaoli Wang, Qingjun Wu, Wen Xie, Youjun Zhang

**Affiliations:** 1 Department of Plant Protection, Institute of Vegetables and Flowers, Chinese Academy of Agricultural Sciences, Beijing 100081, China; jixingxia@126.com (J.X.); xhf846664635@163.com (H.X.); yangzhezhong_1988@126.com (Z.Y.); xinyang3856@163.com (X.Y.); guozhaojiang@caas.cn (Z.G.); guolitao@caas.cn (L.G.); xiaodong0429@126.com (X.S.); wangshaoli@caas.cn (S.W.); wuqingjun@caas.cn (Q.W.); xiewen@caas.cn (W.X.); 2 Key Laboratory of Bio-Pesticide Innovation and Application of Guangdong Province, South China Agricultural University, Guangzhou 510642, China; panhuipeng@scau.edu.cn; 3 Key Laboratory of Molecular Biology of Heilongjiang Province, College of Life Sciences, Heilongjiang University, Harbin 150080, China; yangfshan@126.com

**Keywords:** carboxylesterases, *Bemisia tabaci*, expression profile, imidacloprid resistance

## Abstract

The whitefly (*Bemisia tabaci*), an important invasive pest that causes severe damage to crops worldwide, has developed resistance to a variety of insecticides. Carboxylesterases (COEs) are important multifunctional enzymes involved in the growth, development, and xenobiotic metabolism of insects. However, systematic studies on the COEs of *B. tabaci* are scarce. Here, 42 putative COEs in different functional categories were identified in the Mediterranean species of *B. tabaci* (*B. tabaci* MED) based on a genome database and neighbor-joining phylogeny. The expression patterns of the COEs were affected by the development of *B. tabaci*. The expression levels of six COEs were positively correlated with the concentration of imidacloprid to which *B. tabaci* adults were exposed. The mortality of *B. tabaci* MED adults fed dsBTbe5 (67.5%) and dsBTjhe2 (58.4%) was significantly higher than the adults fed dsEGFP (41.1%) when treated with imidacloprid. Our results provide a basis for functional research on COEs in *B. tabaci* and provide new insight into the imidacloprid resistance of *B. tabaci*.

## 1. Introduction

Carboxylesterases (COEs) are multi-gene superfamily enzymes with an α/β-hydrolase fold that can hydrolyze carboxyl esters into corresponding alcohols and acids [1], and are widely found in animals [2] (including insects [3]), plants [4], and microorganisms [5]. COEs have a wide range of biological functions; they are not only involved in the important process of nerve development, but also participate in the degradation of hormones and pheromones [6]. More importantly, as important metabolic enzymes, COEs participate in the detoxification of ester-containing xenobiotics such as drugs, insecticides, and environmental toxicants [7].

In insects, studies on COEs have mainly focused on their roles in insecticide resistance. The mutation of COE coding sequences is related to organophosphorus resistance in Hemiptera (*Bemisia tabaci* Middle East-Asia Minor 1 (MEAM1) [8] and *Aphis gossypii* [9]), Lepidoptera (*Plodia interpunctella* [10]), Hymenoptera (*Anisopteromalus calandrae* [11]), and Diptera (*Lucilia cuprina* [12] and *Musca domestica* [13]). The overexpression of COEs caused by gene amplification or transcriptional upregulation is involved in insecticide resistance in Hemiptera (*B. tabaci* MEAM1 [8], *Nilaparvata lugens* [14], and *A. gossypii* [15]), Lepidoptera (*Helicoverpa armigera* [16]), Hymenoptera (*Habrobracon hebetor* [10]), and Diptera (*Culex quinquefasciatus* [17]). The induction of COE gene expression by insecticides is considered an important cause of insecticide resistance in Hemiptera (*A. gossypii* Glover [18]), Coleoptera (*Leptinotarsa decemlineata* [19]), Diptera (*Aedes aegypti* [20]), and Arachnida (*Tetranychus cinnabarinus* [21]). COEs frequently increase insecticide resistance through gene coding sequence mutations, constitutive overexpression, inductive expression, or a combination of these mechanisms [10].

The whitefly, *B. tabaci* (Gennadius), is one of the 100 most catastrophic invasive species in the world [22]. This typical phloem sap-feeding insect causes severe crop reductions by directly feeding on phloem, transmitting various plant viruses, and excreting honeydew. Over 600 different plant species, including crops and ornamentals, have been documented as hosts of *B. tabaci* [23]. *B. tabaci* is a species complex that includes at least 30 cryptic species [24]. Among these species, the MEAM1 species (formerly biotype B) and Mediterranean species (MED, formerly biotype Q) are considered the most invasive and destructive cryptic species [25]. In most parts of China, the previously dominant *B. tabaci* MEAM1 has been replaced by *B. tabaci* MED due to the overuse of insecticides [26,27,28].

Neonicotinoid insecticides are an important class of chemical insecticides that are used worldwide because of their high toxicity to a range of important pests [29,30,31]. With increased neonicotinoid insecticide use, insects inevitably develop resistance [31]. *B. tabaci* was the first insect to develop resistance to imidacloprid [32], and *B. tabaci* resistance to neonicotinoid insecticides (including imidacloprid) has been reported globally [27,33,34,35,36,37]. The detoxification enzymes glutathione S-transferase and cytochrome P450 monooxygenases are involved in the resistance of *B. tabaci* to imidacloprid [34,38,39,40,41,42]. However, whether COEs contribute to *B. tabaci* MED tolerance of imidacloprid has not been investigated.

Recently, our group sequenced the transcriptome and genome of *B. tabaci* MED [43,44], providing a solid foundation for a comprehensive study of whitefly COE family genes at the genome level. In the current study, we provide genome-wide annotation and classification of COEs in the *B. tabaci* MED genome by constructing a phylogenetic tree with homologous genes from *Apis mellifera* and *Drosophila melanogaster*. We then characterized the expression patterns of COE genes affected by developmental stage and investigated the expression profiles of the COE genes in response to imidacloprid challenge. Finally, RNA interference (RNAi) was used to determine which COEs are involved in *B. tabaci* MED defense against imidacloprid.

## 2. Results

### 2.1. Identification of COEs in the Genome of B. tabaci MED

**Figure 1:**
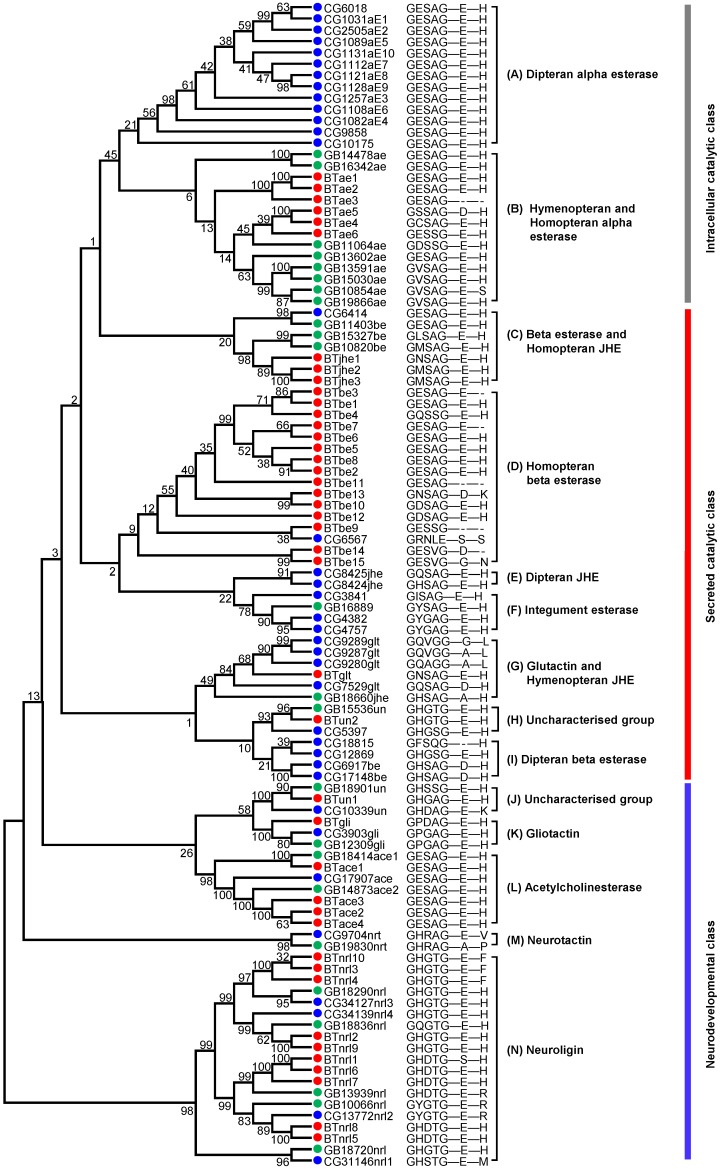
Phylogenetic tree of COEs in *B. tabaci* MED. A total of 103 COE amino acid sequences were used in the phylogenetic analysis. The phylogenetic tree was constructed using MEGA 6 with the neighbor-joining (NJ) method based on the Jones–Taylor–Thornton (JTT) model with a uniform substitution rate. Bootstrap values shown at branch points are expressed as percentages calculated from 1000 replicates. The catalytic residues and GXSXG consensus sequence around serine active site are shown at the right of the phylogenetic tree. *B. tabaci* MED (red circles); *D. melanogaster* (blue circles); *A. mellifera* (green circles).

Through genomic analysis and transcriptome correction, a total of 42 putative COEs were identified in the genome of *B. tabaci* MED (Table S1). These COEs were located on 34 scaffolds, among which scaffolds 673 and 4145 each contained two COEs and scaffolds 39, 436, and 601 each contained three COEs (Table S1). The lengths of COE coding proteins ranged from 302 to 932 amino acids (Table S1). A neighbor-joining (NJ) phylogenetic tree of COEs was constructed with MEGA 6.0 using multiple alignments of amino acid sequences from *B. tabaci* MED, *A. mellifera*, and *D. melanogaster* to survey gene phylogenetic relationships. The phylogenetic tree divided the insect COEs into nine clades that were clustered into three groups: the intracellular catalytic class, secreted catalytic class, and neurodevelopmental class (Figure 1, Table S1). Six COEs belonged to alpha esterase, which was the only clade in the intracellular catalytic class. Seventeen COEs were involved in secreted catalytic processes: fifteen beta esterases, three juvenile hormone esterases (JHEs), one glutactin, and one uncharacterized esterase. The neurodevelopmental class included 16 COEs: ten neuroligins, four acetylcholinesterases, one gliotactin, and one uncharacterized esterase. *Acyrthosiphon pisum* and *B. tabaci* contained more beta esterase genes but fewer alpha esterase genes than the other five insects. *B. tabaci* contained the largest number of neuroligin genes (Table 1).

### 2.2. Expression Profiling of B. tabaci MED COE Genes

**Figure 2:**
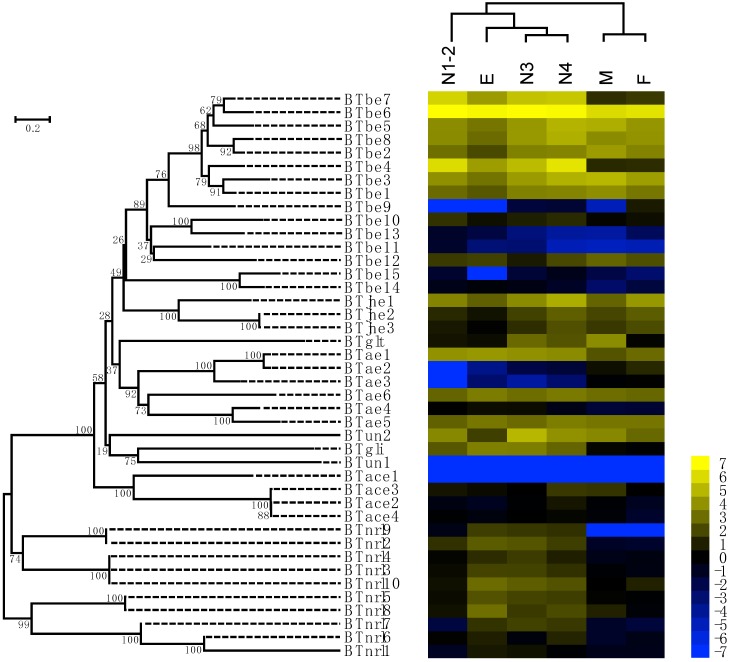
Expression of COE genes among *B. tabaci* MED developmental stages. The phylogenetic tree on the left shows the phylogenetic relationships of COE in *B. tabaci* MED. A total of 42 COE amino acid sequences of *B. tabaci* MED were aligned by MUSCLE, and the phylogenetic tree was constructed by MEGA 6.0 using the NJ method based on the JTT model with a uniform substitution rate. Bootstrap values displayed at branch points are expressed as percentages of 1000 replicates. The color scale is displayed on the right side; yellow represents higher expression values (log2-transformed FPKM values), while blue represents lower expression values (log2-transformed FPKM values). E, Egg; N1-2, 1st- and 2nd-instar nymphs; N3, 3rd-instar nymph; N4, 4th-instar nymph; M, Male; F, Female.

COE family genes are involved in multiple processes of insect growth and development; thus, their expression levels may vary among developmental stages. To determine the expression profiles of COEs across different developmental stages of *B. tabaci* MED, we extracted the total RNA from eggs, 1st-2nd-instar nymphs, 3rd-instar nymphs, 4th-instar nymphs, and adults. COE expression levels were examined using RNA-seq. The log2-transformed expression values (FPKM—fragments per kilobase of transcript per million fragments mapped) of COE genes are shown in Figure 2 (Table S3). Hierarchical clustering was carried out using Gene Cluster 3.0 with the centroid linkage method (Figure 2). In the hierarchical clustering results, 3rd- and 4th-instar nymphs first clustered together and then clustered with eggs and 1st-2nd-instar nymphs. Males and females first clustered together and then clustered with other developmental stages.

*BTbe6* was highly expressed at all stages of development, while *BTun1* and *BTace1* were largely undetectable in transcriptional analysis. Examination of the secreted catalytic class showed that *BTbe7* expression was significantly lower in adults than at other stages. *BTbe4* was downregulated in male and female adults but highly expressed in 1st-2nd-instar nymphs and 4th-instar nymphs. *BTbe9* expression was significantly higher in female adults and lower in eggs, males, and 1st-2nd-instar nymphs. The *BTbe15* had significantly lower expression in eggs than at other stages. *BTjhe* genes were most highly expressed in 4th-instar nymphs, followed by female adults. *BTun2* exhibited the highest expression in 3rd-instar nymphs and lowest expression in eggs. *BTglt* was upregulated in male adults. In the intracellular catalytic class, *BTae1* was downregulated while *BTae2* and *BTae3* were upregulated in male and female adults. In the neurodevelopmental class, most of the *BTnrl* genes had the highest expression level in eggs, except for *BTnrl3*, *BTnrl6*, and *BTnrl7*, and all *BTnrl* genes were expressed at low levels in male and female adults. *BTace* genes except for *BTace1* were more highly expressed in 4th-instar nymphs than at the other stages.

**Figure 3:**
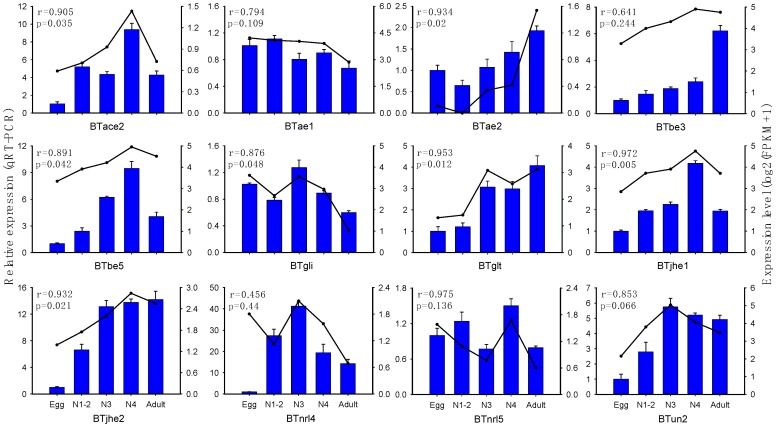
qRT-PCR-based expression profiling of COE genes at different stages of *B. tabaci* MED development. Total RNA was extracted during individual developmental stages of *B. tabaci* MED and used to analyze the expression levels of COEs with qRT-PCR. FPKM values were used to determine the transcript levels of COEs with RNA-seq, and the results are expressed in the form of log2(FPKM+1). Correlation between RNA-seq and qRT-PCR were tested using SPSS 23 with Pearson and two-tailed test, r, Pearson correlation; *p*, significant. E, Egg; N1-2, 1st- and 2nd-instar nymphs; N3, 3rd-instar nymph; N4, 4th-instar nymph; A, Adult.

In order to verify the expression profile data obtained from the transcriptome, qRT-PCR was performed to analyze the expression of 12 COEs, which covered each of the COE clades, at different stages of *B. tabaci* MED development. The expression levels of 7 of the 12 COEs obtained by qRT-PCR were significantly consistent with the FPKM expression pattern (*p* < 0.05) (Figure 3).

### 2.3. Responses of COE Expression to Imidacloprid

**Figure 4:**
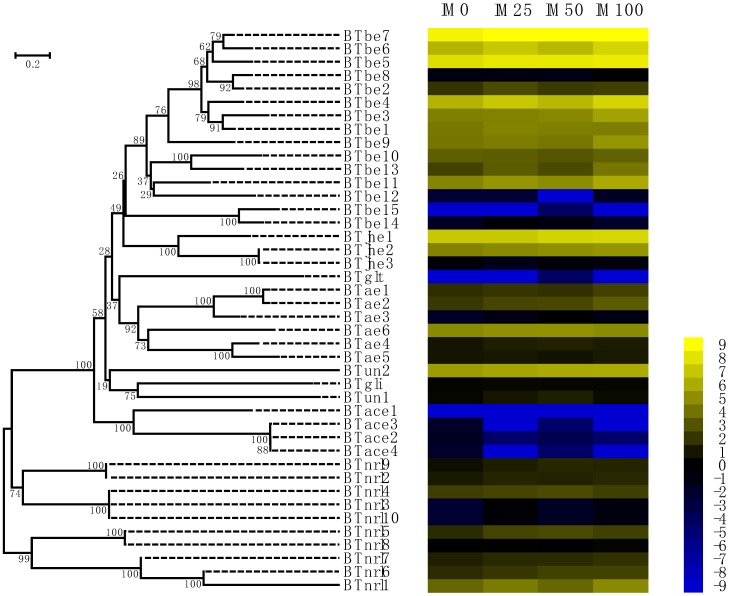
Expression of COE genes in *B. tabaci* MED adults treated with an imidacloprid gradient. A total of 14 COE amino acid sequences of *B. tabaci* MED were aligned by MUSCLE, and the phylogenetic tree was constructed by MEGA 6.0 using the NJ method based on the JTT model with a uniform substitution rate. Bootstrap values displayed at branch points are expressed as percentages of 1000 replicates. The color scale is displayed on the right side; yellow represents higher expression values (log2-transformed FPKM values), while blue represents lower expression values (log2-transformed FPKM values). IM0, whitefly treated with water; IM25, whitefly treated with 25 mg/L imidacloprid; IM50, whitefly treated with 50 mg/L imidacloprid; IM100, whitefly treated with 100 mg/L imidacloprid.

To investigate the tolerance of *B. tabaci* to imidacloprid challenge, we analyzed the expression profiles of all COEs in response to imidacloprid using the leaf-dip bioassay method with imidacloprid concentrations of 0 (control), 25, 50, and 100 mg/L [44]. The expression of most of COE genes (except for eight COE genes of the beta esterase clades (*BTbe8*, *BTbe10*, *BTbe12*, *BTbe14*, and *BTbe15*), all four acetylcholinesterases, and *BTjhe3*, *BTae5*, *BTglt*, and *BTnrl8*) was induced by imidacloprid, and the results are shown in Table S4. The expression levels of *BTbe5, BTbe3, BTjhe1, BTjhe2, BTae2*, and *BTun2* induced by 100 mg/L imidacloprid were higher than those induced by 50 mg/L imidacloprid, followed by 25 mg/L imidacloprid induction. The expression levels of these genes were lowest in the control group with no imidacloprid (Figure 4, Table S4). The COE genes whose expression levels were positively correlated with imidacloprid concentration were used for subsequent study, as these genes are more likely to participate in whitefly resistance to imidacloprid.

### 2.4. COEs Involved in the Imidacloprid Resistance of B. tabaci MED

**Figure 5:**
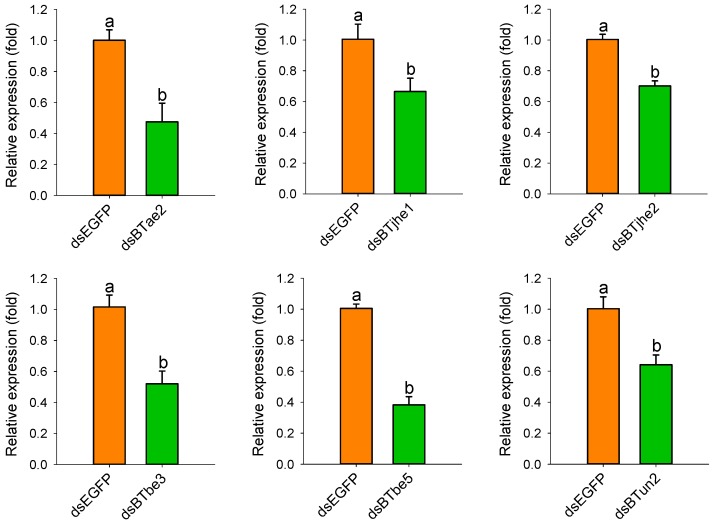
Silencing of COE gene expression by oral feeding of dsRNA. Suppression of COE gene expression after *B. tabaci* MED adults were fed dsRNA for 48 h, with adults fed dsEGFP as a control. The expression of COE genes was detected by qRT-PCR with *EF1-α* and *RPL29* as internal reference genes. Different letters above the bars indicate significant differences between treatments (*p* < 0.05; Holm–Sidak test; *n* = 3).

**Figure 6:**
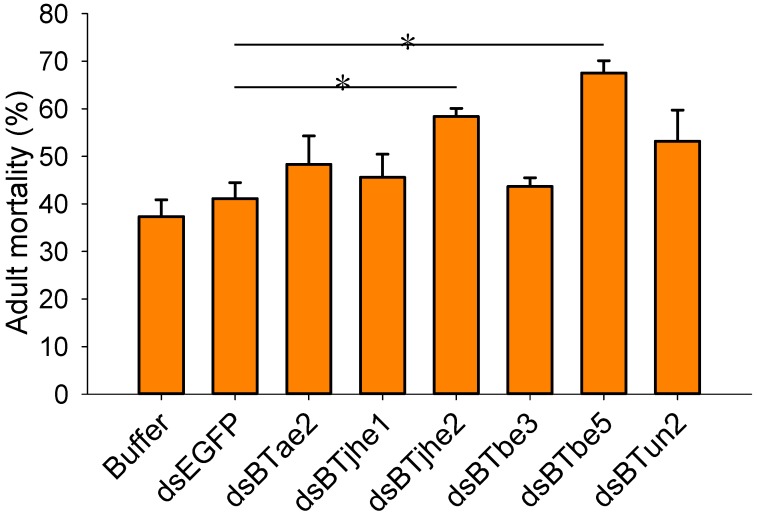
Effect of silencing COE genes on *B. tabaci* susceptibility to imidacloprid. Susceptibility to imidacloprid in *B. tabaci* adults fed buffer (without dsRNA), dsEGFP, or dsRNA targeting COE genes was tested using feeding chambers. Asterisks indicate significant differences between treatments (dsRNA) and the control (dsEGFP) (*p* < 0.05; Holm–Sidak test; *n* = 3).

To determine whether the COEs mentioned above are related to the imidacloprid resistance of *B. tabaci* MED, RNAi was performed to knock down the expression of these genes by feeding dsRNA of COEs to *B. tabaci* adults, and a bioassay was used to assess mortality. The expression levels of COEs in *B. tabaci* fed COE dsRNA for 48 h were significantly lower than those in adults fed enhanced green fluorescent protein (EGFP) dsRNA (*p* < 0.05; *n* = 3; Figure 5). The bioassay results showed that there was no significant difference in the mortality of *B. tabaci* adults fed buffer (without dsRNA) (37.3%) or dsEGFP (41.1%) when the adults were treated with imidacloprid at 50 mg/L (*p* < 0.05; *n* = 3; Figure 6). The mortality of *B. tabaci* MED adults fed dsRNA of *BTbe5* (67.5%) and *BTjhe2* (58.4%) was significantly higher than that of adults fed dsRNA of *EGFP* (41.1%) when treated with imidacloprid (*p* < 0.05; *n* = 3; Figure 6).

## 3. Discussion

COE genes have multiple functions in insects, including neurogenesis, developmental regulation, xenobiotic metabolism, and insecticide detoxification [45]. *B. tabaci* is a globally invasive pest with a wide host adaptability range. A large number of chemical pesticides are used worldwide for the control of *B. tabaci*, resulting in the resistance of *B. tabaci* to insecticides. A comprehensive understanding of *B. tabaci* COE genes will contribute to studies of the physiology, host adaptability, and insecticide resistance of *B. tabaci*. In this study, 42 putative COEs were identified in the *B. tabaci* MED genome, and the expression patterns of the COE genes among *B. tabaci* MED developmental stages were detected from the transcriptome. Expression profile analysis of COEs in *B. tabaci* adults that were treated with imidacloprid at different concentrations and RNAi studies revealed that two COE genes (*BTbe5* and *BTjhe2*) were involved in *B. tabaci* MED imidacloprid resistance.

In the current study, we found that the hemipteran insects *B. tabaci* MED and *A. pisum* had more beta esterase genes but fewer alpha esterase genes than other insects. The alpha esterase genes belong to the intracellular catalytic class of COEs and participate in insect xenobiotic detoxication [6,46,47]. Previous studies have shown that beta esterases have multiple functions, especially in the metabolism of xenobiotics and insecticides [6,17,48]. Some genes in the beta esterase subfamily of hemipteran insects may perform functions similar to those of alpha esterase subfamily genes in other insects, resulting in a decrease in the number of alpha esterase subfamily genes in *B. tabaci* MED. We also found that *B. tabaci* had more *nrl* genes than other insects. *nrl* genes are essential for establishing and remodeling central nervous system synapses [49,50]. Whether the expansion of the *nrl* genes in *B. tabaci* MED is related to its developmental characteristics or the evolution of new biological functions for some of these genes needs to be further investigated.

The expression profile of the COEs at different developmental stages of *B. tabaci* MED showed that adult stages clustered together, while eggs and nymphal stages were clustered together. The COE genes of insects participate in many processes of growth and development. Eggs and nymphs of whiteflies are in the process of rapid growth and development, while adults have completed their development. Therefore, the expression of COEs in *B. tabaci* MED was clustered as described above. The ecdysone and JH in insect hemolymph regulate insect growth and development in a complex manner [51]. In numerous insects, JH esterase (JHE) plays critical roles in the metabolism of JH by hydrolysis of methyl ester, which is considered the principal pathway for the degradation of JH, and is involved in insect development, metamorphosis, diapause, and reproduction [52]. We detected high expression of JHEs in the 4th-instar nymphs of whiteflies. A previous study indicated that the JHE-like gene (Px004817) of *Plutella xylostella* is highly expressed in 4th-instar larvae and participates in the induction of larval-to-pupal metamorphosis [53]. JHE genes likely participate in whitefly development from nymphs to adults. The *BTgli* and *BTnrls* genes, which belong to the neurodevelopment clade, were expressed at low levels in the adult stage, and most of these genes were highly expressed in the egg stage. This difference may be related to the developmental characteristics of *B. tabaci*. During development from the egg to the nymph, whiteflies develop a complex nervous system; therefore, high expression levels of genes involved in neural development are required. In contrast, at the adult stage, the nervous system is fully developed, and as a result, the expression levels of genes related to nerve development will be reduced. RNA-seq and qRT-PCR are the two main methods for gene expression analysis. Previous studies showed that the correlation of ABC transporter expression between RNA-seq and qRT-PCR was approximately 0.684 [44], and 50% (6/12 genes) [43] of amino acid/auxin permease (AAAP) transporter genes showed high consistency between these two methods. In this study, we found that 58% (7/12 genes) of COE genes showed highly similar patterns between the two methods, indicating that our RNA-seq results effectively reflect the expression level of the genes.

The induction of gene expression represents adaptive plasticity between energy conservation and survival in rapidly changing environments [54,55,56]. Our expression profiles of COEs in response to imidacloprid challenge in *B. tabaci* MED showed that most COEs can be induced by imidacloprid, and the expression levels of six COE genes were positively correlated with the concentration of imidacloprid applied. COEs are among the most important detoxification enzyme systems that participate in the metabolic detoxification of xenobiotics in insects. COEs, which increased in expression with increasing imidacloprid concentration, are more likely to be involved in the resistance of *B. tabaci*. Alpha esterases and beta esterases are widely recognized as major COE clades involved in the detoxification of xenobiotics and lipids [7]. *BTbe3, BTbe5*, and *BTae2* were located in these clades, suggesting that these genes play a potentially important role in metabolizing insecticides in *B. tabaci*. JHE genes are mainly involved in the regulation of hormone metabolism, growth, and development [52]. Whether *B. tabaci* JHE genes participate in insecticide resistance requires verification by subsequent RNAi experiments.

Neonicotinoid insecticides are important chemical pesticides that are widely used in the control of agricultural pests worldwide [29,30,31]. In the past few decades, *B. tabaci* has developed high levels of resistance to neonicotinoids in the field [27,33,34,35,36,37]. Previous studies have shown that the resistance of *B. tabaci* against neonicotinoid insecticides is mainly related to detoxification [7,8,34,38,40,57,58]. The enhanced activity of cytochrome P450 monooxygenases (P450s) is generally recognized as the major mechanism of resistance to imidacloprid in *B. tabaci* [34,38,39]. The overexpression of the P450 genes *CYP6CM1* and *CYP4C64* has been found to be involved in imidacloprid resistance in field *B. tabaci* [57]. Silencing the *GSTd7* gene increased the mortality of whiteflies exposed to imidacloprid [41], and knockdown of *GST14* significantly increased the mortality of thiamethoxam-treated *B. tabaci* MED [40]. Recently, an ATP-binding cassette transporter, ABCG3, was shown to be involved in the resistance of *B. tabaci* to imidacloprid, and silencing the *ABCG3* gene by RNAi increased the lethality of imidacloprid to adults of *B. tabaci* MED [58]. Previous transcriptome analysis revealed that the expression level of six COE genes of *B. tabaci* MED corresponded to the amount of imidacloprid to which individuals were exposed. We silenced COE gene expression using oral feeding of dsRNA and found that knockdown of the *BTbe5* and *BTjhe2* genes significantly increased the lethal effect of imidacloprid, indicating that these two genes are involved in the resistance of *B. tabaci* MED to imidacloprid. In insects, the beta esterase clade genes are recognized as important detoxification enzymes involved in resistance to organophosphorus insecticides [17,59,60]. In this study, we found that *BTbe5* was involved in the imidacloprid resistance of *B. tabaci* MED. The main function of JHE genes is to regulate the growth and development of insects. *B. tabaci* adults have completed their growth and development. The expression of the JHE genes in the adult stage indicates that these genes may have other biological functions. In this study, we found that a JHE gene participated in the resistance of adult *B. tabaci* to imidacloprid.

In summary, all COE family genes were identified in *B. tabaci* MED, and the expression profiles of COEs at different developmental stages of *B. tabaci* were analyzed. Moreover, two COE genes, *BTbe5* and *BTjhe2*, were found to facilitate *B. tabaci* MED resistance to imidacloprid. The present results will contribute to functional research on COEs, enrich our understanding of the resistance mechanism of *B. tabaci* MED to imidacloprid, and aid in the development of a management strategy for *B. tabaci*.

## 4. Materials and Methods

### 4.1. Insect Strain

The colony of *B. tabaci* MED was collected on poinsettia (*Euphorbia pulcherrima* Wild. ex Kl.) in 2009 in Beijing. It was transferred and continuously maintained on cotton (*Gossypium herbaceum* L. cv. E-Mian 24) without exposure to chemical pesticides in a glasshouse at 27 ± 1 °C, with a relative humidity (RH) of 70% ± 10% and L16:D8 photoperiod. The purity of the colony was monitored by sequencing a fragment of the mitochondrial cytochrome oxidase I (mtCOI) gene every three to five generations [26].

### 4.2. De Novo Identification of COE Genes

To identify the putative COEs in *B. tabaci* MED, the predicted proteins containing the conserved functional domain of COE defined by the Pfam hidden Markov model (HMM) profile *COesterase* (PF00135) were identified in the *B. tabaci* MED genome-predicted protein data set [43] using HMMER (v.3.01) [61]. The COE amino acid sequences of *A. mellifera* and *D. melanogaster* downloaded from the NCBI (https://www.ncbi.nlm.nih.gov/genome/?term=Apis+mellifera (accessed on 12 March 2018)) and FlyBase (http://flybase.org/ (accessed on 12 March 2018)) were used as queries to search against the *B. tabaci* MED genome to ensure that all the COEs had been identified. A total of 42 putative *B. tabaci* COE genes were confirmed by blasting against the NR database with the BLASTX program on the NCBI website (http://blast.ncbi.nlm.nih.gov/ (accessed on 16 March 2018)). Then, the putative COEs were manually corrected by comparison with assembled expressed sequence tags (ESTs) [44].

### 4.3. Phylogenetic Analysis of B. tabaci MED COE Genes

To comprehensively annotate and systematically classify the COE genes of *B. tabaci* MED, a phylogenetic tree was constructed based on the putative COE genes of *B. tabaci* MED and COE amino acid sequences from *D. melanogaster* and *A. mellifera*. All the selected COE amino acid sequences were aligned using MUSCLE, a module of MEGA 6 (http://www.megasoftware.net/ (accessed on 6 May 2018)) [62]. A phylogenetic tree was generated using the neighbor-joining (NJ) method based on the Jones–Taylor–Thornton (JTT) model with a uniform substitution rate combined with pairwise deletion and 1000 bootstrap replicates.

### 4.4. RNA-Seq Analysis

RNA-seq libraries for different developmental stages of *B. tabaci* MED and imidacloprid-treated adult *B. tabaci* MED were obtained from our previous report [44]. Trimmomatic was used to filter the transcriptome datasets [63], and the clean data were mapped to the *B. tabaci* MED genome with TopHat software [43,64]. The fragments per kilobase of transcript per million fragments mapped (FPKM) value was calculated using Cufflinks to estimate the expression level of each predicted transcript [65].

### 4.5. RNA Isolation and cDNA Synthesis

Samples were collected from *B. tabaci* MED at different developmental stages (eggs, first- to fourth-instar nymphs, and newly emerged (0- to 2-day-old) adults). Total RNA was extracted from each sample using TRIzol reagent (Invitrogen, Carlsbad, CA, USA) following the manufacturer’s instructions. RNA quality was evaluated by agarose gel electrophoresis, and the total RNA was quantified using a spectrophotometer (NanoDrop 2000c, Thermo Fisher Scientific Inc., Waltham, MA, USA). The RNA was reverse-transcribed to cDNA with a PrimeScript RT kit (Perfect Real Time) (TaKaRa, Dalian, China) for qRT-PCR analysis and a PrimeScript™ II 1st strand cDNA synthesis kit (TaKaRa, Dalian, China) for COE double-strand RNA (dsRNA) synthesis. The cDNA was stored at −80 °C for later use.

### 4.6. Quantitative Real-Time Polymerase Chain Reaction (qRT-PCR) Analysis

The specific primers for COEs of *B. tabaci* MED were designed using Primer Premier 5.0 and used to verify the expression levels of COEs by qRT-PCR (Table S2). qRT-PCR was performed using an ABI 7500 system (Applied Biosystems) with the 25-μL reaction containing 0.5 μL of each specific primer, 0.5 μL of 50 × ROX reference dye (TIANGEN, Beijing, China), 1 μL of cDNA template, 10 μL of ddH_2_O, and 12.5 μL of 2 × SuperReal PreMix Plus (SYBR Green) (TIANGEN, Beijing, China). The qRT-PCR programme was as follows: 95 °C for 10 min (initial denaturation), followed by 40 cycles of 95 °C for 5 s (denaturation), 60 °C for 15 s (annealing), and 72 °C for 35 s (elongation). Only the qRT-PCR primers with 90–110% amplification efficiencies were used for the subsequent data analysis.

Relative expression levels were quantified using the 2^−ΔΔ*Ct*^ method [66]. The geometric mean of the reference genes 60S ribosomal protein L29 (*RPL29*) (GenBank accession no. EE596314) and elongation factor 1 alpha (*EF1-α*) (GenBank accession no. EE600682) was used to normalize the expression of target genes [67,68]. Three biological replicates and four technical replicates were performed for each sample. One-way analysis of variance (ANOVA) (SPSS 23) was used to detect significant differences between samples.

### 4.7. dsRNA Synthesis and RNAi Assays

*BTbe5, BTbe3, BTTjhe1, BTjhe2, BTae2*, and *BTun2* were selected for RNAi because their expression was positively correlated with the imidacloprid-induced concentration. The dsRNA primers of *BTbe5, BTbe3, BTjhe1, BTjhe2, BTae2, BTun2*, and *EGFP* (GenBank: KC896843) with the T7 promoter sequence were designed using Primer Premier 5.0 to clone partial cDNA of those genes (Table S2). The dsRNA of *BTbe5, BTbe3, BTjhe1, BTjhe2, BTae2, BTun2*, and *EGFP* were synthesized using the T7 Ribomax™ Express RNAi System (Promega, Madison, WI, USA). The quality of dsRNA was evaluated by gel electrophoresis, and the dsRNA was quantified using a NanoDrop spectrophotometer.

Knockdown of *BTbe5, BTbe3, BTjhe1, BTjhe2, BTae2,* and *BTun2* genes was performed by orally feeding dsRNA to *B. tabaci* MED adults in feeding chambers. The feeding chambers contained 0.2 mL of diet solution, which contained 30% sucrose, 5% yeast extract (weight/volume), with 0.5 μg/μL dsBTbe5, dsBTbe3, dsBTjhe1, dsBTjhe2, dsBTae2, and dsBTun2 [69]. Approximately 60 newly emerged (<2 days old) *B. tabaci* MED adults (mixed sexes) were released into the feeding chambers, and were kept at 25 °C, 80% RH, and an L14:D10 photoperiod. The effectiveness of RNAi was determined by qRT-PCR using cDNA synthesized from isolated total adult RNA after 2 days of feeding. Each RNAi treatment was repeated three times.

Bioassays were performed using feeding chambers for 24 h. The living whitefly adults fed dsRNA for 48 h were transferred to a new feeding chamber containing 50 mg/L imidacloprid in a diet solution. Bioassays were performed with approximately 40 living whitefly adults per treatment, and each bioassay was replicated three times. Significant differences in the bioassays were determined using SPSS 23 with one-way ANOVA and Holm–Sidak test (overall significance level = 0.05).

## Figures and Tables

**Table 1:** The distribution of carboxylesterase (COE) genes in *B. tabaci* MED and other insects

Class/Clades	*B. mori*	*D. melanogaster*	*Ap. mellifera*	*An. gambiae*	*T. castaneum*	*A. pisum*	*B. tabaci*
Intracellular catalytic class							
α-esterase	55	13	8	16	26	5	6
secreted catalytic class							
JHE	4	2	1	9	1	0	3
integument esterase	2	3	1	0	2	0	0
β-esterase	2	6	3	5	8	15	15
uncharacterized	1	1	1	1	1	1	1
glutactin	0	4	0	9	0	0	1
neurodevelopmental class							
AChE	2	1	2	2	2	2	4
uncharacterized	1	1	1	1	1	1	1
gliotactin	1	1	1	1	1	1	1
neuroligin	6	4	5	5	4	3	10
neurotactin	2	1	1	2	2	0	0
total	76	37	24	51	48	28	42

*A. pisum*, *Acyrthosiphon pisum*; *An. gambiae*, *Anopheles gambiae*; *Ap. mellifera*, *Apis mellifera*; *B. mori*, *Bombyx mori*; *B. tabaci*, *Bemisia tabaci* MED; *D. melanogaster*, *Drosophila melanogaster*; *T. castaneum*, *Tribolium castaneum*. AChE: acetylcholinesterase; JHE, juvenile hormone esterase.

## Supplementary Materials

Supplementary materials can be found at https://www.mdpi.com/1422-0067/20/20/4973/s1. Table S1 Identification of COE genes in the *B. tabaci* MED genome. Table S2. Primers used to study COEs. Table S3. FPKM values of the COE genes affected by developmental stages of *B. tabaci* MED. Table S4. FPKM values from transcriptome data of COE genes in *B. tabaci* MED adults treated with an imidacloprid gradient.

## Abbreviations

COE	Carboxylesterase
RNAi	RNA interference
FPKM	Fragments per kilobase of transcript per million fragments mapped
NJ	Neighbor-joining
JTT	Jones–Taylor–Thornton
dsRNA	Double-strand RNA
EGFP	Enhanced green fluorescent protein
JH	Juvenile hormone
JHE	Juvenile hormone esterase
qRT-PCR	Quantitative real-time polymerase chain reaction
AAAP	Amino acid/auxin permease
P450	Cytochrome P450 monooxygenase
GST	Glutathione S-transferase
ABC	ATP-binding cassette transporter
mtCOI	Mitochondrial cytochrome oxidase I
HMM	Hidden Markov model
NCBI	National Center for Biotechnology Information
EST	Expressed sequence tag
