# Supplementary material for: Genome-Wide Analysis of Carboxylesterases (COEs) in the Whitefly, Bemisia tabaci (Gennadius)
Source: Int J Mol Sci. 2019 Oct 9;20(20):4973. doi: 10.3390/ijms20204973 (PMC6829539; doi:10.3390/ijms20204973)
Supplement: Supplementary file 1 [file ijms-20-04973-s001.zip › Supplementary Materials.docx]

**Table S1** Identification of COE genes in the *B. tabaci* MED genome.

| **Class/clades** | **Gene ID** | **Gene** | **AA** | **Scaffold** | **Orientation** | **Blast hit** | **Species** | **E-value** | **Identity** |
| --- | --- | --- | --- | --- | --- | --- | --- | --- | --- |
| Alpha esterase | BTA023710.4 | *BTae1* | 551 | 55 | + | [XP_018899525.1](https://www.ncbi.nlm.nih.gov/protein/XP_018899525.1?report=genbank&log$=prottop&blast_rank=1&RID=4PF5AWMM01R) | *B． tabaci* | 0 | 99% |
|  | BTA005477.2 | *BTae2* | 552 | 1532 | - | [XP_018899525.1](https://www.ncbi.nlm.nih.gov/protein/XP_018899525.1?report=genbank&log$=prottop&blast_rank=1&RID=4R5D4MPT014) | *B． tabaci* | 0 | 100% |
|  | BTA005478.1 | *BTae3* | 340 | 40 | - | [XP_018909433.1](https://www.ncbi.nlm.nih.gov/protein/XP_018909433.1?report=genbank&log$=prottop&blast_rank=1&RID=4NSCDAZP014) | *B． tabaci* | 0 | 99% |
|  | BTA025913.1_2 | *BTae4* | 542 | 673 | + | [XP_018901770.1](https://www.ncbi.nlm.nih.gov/protein/XP_018901770.1?report=genbank&log$=prottop&blast_rank=1&RID=4R6YPTDD015) | *B． tabaci* | 0 | 97% |
|  | BTA014810.1 | *BTae5* | 581 | 3 | - | [XP_018902146.1](https://www.ncbi.nlm.nih.gov/protein/XP_018902146.1?report=genbank&log$=prottop&blast_rank=1&RID=4R7A9W9S014) | *B． tabaci* | 0 | 97% |
|  | BTA025913.1_1 | *BTae6* | 563 | 673 | + | [XP_018901770.1](https://www.ncbi.nlm.nih.gov/protein/XP_018901770.1?report=genbank&log$=prottop&blast_rank=1&RID=4P5SNKM301R) | *B． tabaci* | 0 | 99% |
| Juvenile hormone esterase | BTA010352.1 | *BTjhe1* | 560 | 22 | - | [XP_018911064.1](https://www.ncbi.nlm.nih.gov/protein/XP_018911064.1?report=genbank&log$=prottop&blast_rank=1&RID=4R65GFDS014) | *B． tabaci* | 0 | 100% |
|  | BTA001596.1 | *BTjhe2* | 570 | 112 | + | [AGT57960.1](https://www.ncbi.nlm.nih.gov/protein/AGT57960.1?report=genbank&log$=prottop&blast_rank=2&RID=4R6CNKS6015) | *B． tabaci* | 0 | 99% |
|  | BTA014568.1 | *BTjhe3* | 570 | 296 | + | [AGT57960.1](https://www.ncbi.nlm.nih.gov/protein/AGT57960.1?report=genbank&log$=prottop&blast_rank=2&RID=4R6CNKS6015) | *B． tabaci* | 0 | 99% |
| Beta esterase | BTA020057.1_1 | *BTbe1* | 554 | 4145 | - | [XP_018917976.1](https://www.ncbi.nlm.nih.gov/protein/XP_018917976.1?report=genbank&log$=prottop&blast_rank=1&RID=4PK3ZSJB01R) | *B． tabaci* | 0 | 99% |
|  | BTA020057.1_2 | *BTbe2* | 534 | 4145 | - | [XP_018917636.1](https://www.ncbi.nlm.nih.gov/protein/XP_018917636.1?report=genbank&log$=prottop&blast_rank=1&RID=4PVVPNCJ014) | *B． tabaci* | 0 | 95% |
|  | BTA020653.2_1 | *BTbe3* | 385 | 436 | + | [XP_018917976.1](https://www.ncbi.nlm.nih.gov/protein/XP_018917976.1?report=genbank&log$=prottop&blast_rank=1&RID=4PJEPN7V01R) | *B． tabaci* | 0 | 98% |
|  | BTA024693.2_1 | *BTbe4* | 540 | 601 | + | [XP_018917976.1](https://www.ncbi.nlm.nih.gov/protein/XP_018917976.1?report=genbank&log$=prottop&blast_rank=1&RID=4PDMY701014) | *B． tabaci* | 0 | 78% |
|  | BTA020653.2_3 | *BTbe5* | 599 | 436 | + | [XP_018917733.1](https://www.ncbi.nlm.nih.gov/protein/XP_018917733.1?report=genbank&log$=prottop&blast_rank=1&RID=4PHUDDXF014) | *B． tabaci* | 0 | 91% |
|  | BTA019051.2 | *BTbe6* | 555 | 39 | + | [XP_018910005.1](https://www.ncbi.nlm.nih.gov/protein/XP_018910005.1?report=genbank&log$=prottop&blast_rank=1&RID=4PX3NKYP014) | *B． tabaci* | 0 | 97% |
|  | BTA024693.2_2 | *BTbe7* | 349 | 601 | + | [XP_018910005.1](https://www.ncbi.nlm.nih.gov/protein/XP_018910005.1?report=genbank&log$=prottop&blast_rank=1&RID=4PE0UAY1015) | *B． tabaci* | 0 | 87% |
|  | BTA020653.2_2 | *BTbe8* | 369 | 436 | + | [XP_018917636.1](https://www.ncbi.nlm.nih.gov/protein/XP_018917636.1?report=genbank&log$=prottop&blast_rank=1&RID=4PJ1J91W015) | *B． tabaci* | 0 | 98% |
|  | BTA006966.1 | *BTbe9* | 302 | 1704 | + | [XP_018907125.1](https://www.ncbi.nlm.nih.gov/protein/XP_018907125.1?report=genbank&log$=prottop&blast_rank=1&RID=4R56EFD4014) | *B． tabaci* | 2E-169 | 92% |
|  | BTA023767.1 | *BTbe10* | 551 | 552 | - | [XP_018917942.1](https://www.ncbi.nlm.nih.gov/protein/XP_018917942.1?report=genbank&log$=prottop&blast_rank=1&RID=4PEVJTY101R) | *B． tabaci* | 0 | 100% |
|  | BTA019070.1 | *BTbe11* | 340 | 39 | + | [XP_018902311.1](https://www.ncbi.nlm.nih.gov/protein/XP_018902311.1?report=genbank&log$=prottop&blast_rank=1&RID=4PWBY20A014) | *B． tabaci* | 0 | 98% |
|  | BTA010825.1 | *BTbe12* | 556 | 228 | - | [XP_018910667.1](https://www.ncbi.nlm.nih.gov/protein/XP_018910667.1?report=genbank&log$=prottop&blast_rank=1&RID=4R3SM8NP015) | *B． tabaci* | 0 | 99% |
|  | BTA009097.1 | *BTbe13* | 432 | 20 | - | [XP_018918109.1](https://www.ncbi.nlm.nih.gov/protein/XP_018918109.1?report=genbank&log$=prottop&blast_rank=1&RID=4R51HGW3015) | *B． tabaci* | 0 | 97% |
|  | BTA014018.1 | *BTbe14* | 510 | 284 | + | [XP_018904128.1](https://www.ncbi.nlm.nih.gov/protein/XP_018904128.1?report=genbank&log$=prottop&blast_rank=1&RID=4R1WBP90015) | *B． tabaci* | 0 | 94% |
|  | BTA016353.1 | *BTbe15* | 486 | 330 | - | [XP_018904128.1](https://www.ncbi.nlm.nih.gov/protein/XP_018904128.1?report=genbank&log$=prottop&blast_rank=1&RID=4R08FMH0015) | *B． tabaci* | 0 | 98% |
| Uncharacterised | BTA010451.1 | *BTun2* | 868 | 221 | - | [XP_018899849.1](https://www.ncbi.nlm.nih.gov/protein/XP_018899849.1?report=genbank&log$=prottop&blast_rank=1&RID=4NTD1WZA014) | *B． tabaci* | 0 | 99% |
| Glutactin | BTA013104.1 | *Btglu* | 615 | 2668 | - | [XP_018917075.1](https://www.ncbi.nlm.nih.gov/protein/XP_018917075.1?report=genbank&log$=prottop&blast_rank=1&RID=4R33JP05014) | *B． tabaci* | 0 | 99% |
| Acetylcholinesterae | BTA030002.1 | *BTace1* | 697 | 10 | - | [XP_018895731.1](https://www.ncbi.nlm.nih.gov/protein/XP_018895731.1?report=genbank&log$=prottop&blast_rank=1&RID=4R5Y9ZKT015) | *B． tabaci* | 0 | 100% |
|  | BTA028173.1 | *BTace2* | 730 | 83 | - | [ABV45414.1](https://www.ncbi.nlm.nih.gov/protein/ABV45414.1?report=genbank&log$=prottop&blast_rank=1&RID=4NYP58F301R) | *B． tabaci* | 0 | 100% |
|  | BTA022930.1 | *BTace3* | 730 | 52 | + | [ABV45414.1](https://www.ncbi.nlm.nih.gov/protein/ABV45414.1?report=genbank&log$=prottop&blast_rank=1&RID=4NYP58F301R) | *B． tabaci* | 0 | 100% |
|  | BTA010397.1 | *BTace4* | 730 | 220 | + | [ABV45414.1](https://www.ncbi.nlm.nih.gov/protein/ABV45414.1?report=genbank&log$=prottop&blast_rank=1&RID=4R4J662U015) | *B． tabaci* | 0 | 100% |
| Uncharacterised | BTA030000.1 | *BTun1* | 589 | 15 | + | [XP_018909357.1](https://www.ncbi.nlm.nih.gov/protein/XP_018909357.1?report=genbank&log$=prottop&blast_rank=1&RID=4R5R9BHA015) | *B． tabaci* | 0 | 96% |
| Gliotactin | BTA025668.1 | *BTgli* | 900 | 659 | + | [XP_018906011.1](https://www.ncbi.nlm.nih.gov/protein/XP_018906011.1?report=genbank&log$=prottop&blast_rank=1&RID=4PDD4BJS014) | *B． tabaci* | 0 | 100% |
| Neuroligin | BTA021332.1 | *BTnrl1* | 628 | 4591 | - | [XP_018899662.1](https://www.ncbi.nlm.nih.gov/protein/XP_018899662.1?report=genbank&log$=prottop&blast_rank=1&RID=4PH22WUS01R) | *B． tabaci* | 3E-115 | 98% |
|  | BTA010783.1 | *BTnrl2* | 931 | 227 | + | [XP_018897827.1](https://www.ncbi.nlm.nih.gov/protein/XP_018897827.1?report=genbank&log$=prottop&blast_rank=1&RID=4R493GWW015) | *B． tabaci* | 8E-104 | 100% |
|  | BTA019049.1 | *BTnrl3* | 932 | 39 | + | [XP_018910015.1](https://www.ncbi.nlm.nih.gov/protein/XP_018910015.1?report=genbank&log$=prottop&blast_rank=1&RID=4PXHT05U014) | *B． tabaci* | 0 | 100% |
|  | BTA024690.1 | *BTnrl4* | 929 | 601 | + | [XP_018910015.1](https://www.ncbi.nlm.nih.gov/protein/XP_018910015.1?report=genbank&log$=prottop&blast_rank=1&RID=4PE9WE13015) | *B． tabaci* | 0 | 100% |
|  | BTA028144.1 | *BTnrl5* | 722 | 827 | + | [XP_018898206.1](https://www.ncbi.nlm.nih.gov/protein/XP_018898206.1?report=genbank&log$=prottop&blast_rank=1&RID=4P4FU1ZV014) | *B． tabaci* | 0 | 100% |
|  | BTA023604.1 | *BTnrl6* | 550 | 46 | - | [XP_018899662.1](https://www.ncbi.nlm.nih.gov/protein/XP_018899662.1?report=genbank&log$=prottop&blast_rank=1&RID=4PFJ11XN01R) | *B． tabaci* | 0 | 100% |
|  | BTA023609.1 | *BTnrl7* | 690 | 544 | - | [XP_018899662.1](https://www.ncbi.nlm.nih.gov/protein/XP_018899662.1?report=genbank&log$=prottop&blast_rank=1&RID=4PFARS97014) | *B． tabaci* | 0 | 100% |
|  | BTA021640.1 | *BTnrl8* | 729 | 472 | - | [XP_018898206.1](https://www.ncbi.nlm.nih.gov/protein/XP_018898206.1?report=genbank&log$=prottop&blast_rank=1&RID=4PG50YS901R) | *B． tabaci* | 0 | 99% |
|  | BTA020355.1 | *BTnrl9* | 931 | 425 | + | [XP_018897827.1](https://www.ncbi.nlm.nih.gov/protein/XP_018897827.1?report=genbank&log$=prottop&blast_rank=1&RID=4PJNHG7B015) | *B． tabaci* | 0 | 100% |
|  | BTA016715.1 | *BTnrl10* | 932 | 34 | - | [XP_018910015.1](https://www.ncbi.nlm.nih.gov/protein/XP_018910015.1?report=genbank&log$=prottop&blast_rank=1&RID=4PYF2WY2014) | *B． tabaci* | 2E-57 | 100% |

**Table S2.** Primers used to study COEs.

| **Purpose** | **Primer Name** | **Sequence (5'-3')** |
| --- | --- | --- |
|  | qBTace2-F | GGCGACGAGGTGGAGTATGTG |
|  | qBTace2-R | CCTGTTGGTTTCCCTGTCATTGC |
|  | qBTae1-F | GAGGTAGACGGAGGACTAATTGAGG |
|  | qBTae1-R | CAGTAGGTGGGCGAGCATAGG |
|  | qBTae2-F | CAAAAAGCGTCGAGAAATGG |
|  | qBTae2-R | TCGGCTCCTGAAATAATGGG |
|  | qBTbe3-F | TTTGGCGAGAGTGCTGGAGG |
|  | qBTbe3-R | AGCCGCACAACACAGCCAGA |
|  | qBTbe5-F | CAATCACACCTCCACAGTTGCTTAG |
|  | qBTbe5-R | ATTCAAATCGCCAACAACCCAGTC |
|  | qBTgli-F | CGCTGAATCAAGAAGGGGTTT |
|  | qBTgli-R | GACTGACGAGGAGTTTTACGA |
|  | qBTglt-F | TGGTGCCTCCTGGTCTATTTCATC |
| qRT-PCR analysis | qBTglt-R | TGCGTCTGCTTTGTTTGTAGTGG |
|  | qBTjhe1-F | GCGGCAGCGAACAAATCTCC |
|  | qBTjhe1-R | CCAAATCATCAGCGTGACTTACTCC |
|  | qBTjhe2-F | CGCCCTGCTCTCCAAATAGTTAAAC |
|  | qBTjhe2-R | ACGGTTCGGTTCCTGCCATC |
|  | qBTnrl4-F | TCATCACCACCACCACCATAACG |
|  | qBTnrl4-R | CCACCATTCTCTTGTCGCTTCTTG |
|  | qBTnrl5-F | TACTTCCTCCACTTCCAGCATCAG |
|  | qBTnrl5-R | GCCGCCGACTAAAGGTAATCC |
|  | qBTun2-F | CCTAACTCAACGGCGACCATCTC |
|  | qBTun2-R | CGGCGGCGACGATTCTGC |
|  | dsBTjhe1-F | T7-GATCGGAGTGTGGAATGCTT |
|  | dsBTjhe1-R | T7-GTGGGATTTCTCGACGTTGT |
|  | dsBTjhe2-F | T7-TTAAACTTCTCGCAACGCCT |
|  | dsBTjhe2-R | T7-CTGTAGAGCCCCAGCTCATC |
|  | dsBTbe3-F | T7-TTGGACATTCAAGCTGCAAG |
|  | dsBTbe3-R | T7-AGGGTCTCCACCGAAGTTTT |
| dsRNA synthesis^1^ | dsBTbe5-F | T7-GCCAATCTTGGTTTGAAGGA |
|  | dsBTbe5-R | T7-TAAAAGGGATTTTGGACCCC |
|  | dsBTae2-F | T7-TCACCATAATGACGCCTGAA |
|  | dsBTae2-R | T7-GGCTGAAAAACTCAGGCAAG |
|  | dsBTun2-F | T7-TGCAGTGAAAAACTTGGCAG |
|  | dsBTun2-R | T7-GGACTTGGTTTAGTGGGCAA |
|  | dsEGFP-F | T7-CCACAAGTTCAGCGTGTCCG |
|  | dsEGFP-R | T7-AAGTTCACCTTGATGCCGTTC |

^1^Forward and reverse primers with the T7 RNA polymerase promoter (5’-TAATACGACTCACTATAGGGAGA-3’) at the 5′ end were used to synthetize dsRNA.

**Table S3.** FPKM values of the COE genes affected by developmental stages of *B. tabaci* MED.

| **Gene** | **E**^1^ | **N1-2**^1^ | **N3**^1^ | **N4**^1^ | **M**^1^ | **F**^1^ |
| --- | --- | --- | --- | --- | --- | --- |
| *BTbe7* | 18.9671 | 55.7392 | 40.2366 | 44.2049 | 2.5996 | 3.2036 |
| *BTbe6* | 111.3331 | 138.9867 | 125.0483 | 116.0333 | 64.3248 | 75.4898 |
| *BTbe5* | 9.1532 | 14.1104 | 17.5905 | 29.9778 | 26.7046 | 17.0125 |
| *BTbe8* | 7.7677 | 13.9824 | 17.9206 | 26.998 | 13.8493 | 16.1739 |
| *BTbe2* | 4.139 | 8.1194 | 11.4392 | 12.2824 | 19.4373 | 11.927 |
| *BTbe4* | 19.7175 | 65.0035 | 34.9183 | 76.7091 | 2.3673 | 2.4946 |
| *BTbe3* | 8.7677 | 14.9824 | 18.9206 | 28.998 | 31.8493 | 20.173 |
| *BTbe1* | 5.4141 | 7.5291 | 11.6019 | 12.2722 | 14.814 | 9.2629 |
| *BTbe9* | 0.0001 | 0.0001 | 0.3188 | 0.3605 | 0.0328 | 1.8375 |
| *BTbe10* | 1.5165 | 2.7275 | 1.964 | 2.4153 | 1.2073 | 1.4945 |
| *BTbe13* | 0.2795 | 0.4433 | 0.1006 | 0.0546 | 0.0504 | 0.1781 |
| *BTbe11* | 0.1164 | 0.4062 | 0.1269 | 0.0337 | 0.0295 | 0.0352 |
| *BTbe12* | 3.5486 | 3.1662 | 1.8546 | 4.0588 | 6.699 | 4.6066 |
| *BTbe15* | 0.0001 | 0.3917 | 0.3384 | 0.5483 | 0.2629 | 0.1321 |
| *BTbe14* | 0.7177 | 0.5635 | 0.6064 | 0.4524 | 0.1461 | 0.2994 |
| *BTjhe1* | 6.2194 | 12.1502 | 14.0104 | 25.991 | 6.7114 | 17.479 |
| *BTjhe2* | 1.6118 | 2.3655 | 3.6063 | 6.1631 | 3.9405 | 5.7581 |
| *BTjhe3* | 1.1047 | 1.7858 | 2.5714 | 4.7167 | 3.0868 | 4.3784 |
| *BTglt* | 1.4688 | 1.6497 | 7.4426 | 4.9781 | 14.2064 | 1.2025 |
| *BTae1* | 17.6932 | 15.9711 | 15.3232 | 14.0474 | 4.9679 | 7.5378 |
| *BTae2* | 0.0766 | 0.0001 | 0.2661 | 0.3364 | 1.5203 | 2.2916 |
| *BTae3* | 0.1322 | 0.0001 | 0.0506 | 0.1292 | 0.8652 | 1.0984 |
| *BTae6* | 10.4439 | 6.7818 | 8.0627 | 10.0984 | 8.0591 | 7.1584 |
| *BTae4* | 1.4554 | 1.013 | 1.4324 | 0.5819 | 0.3281 | 0.3683 |
| *BTae5* | 9.543 | 6.3197 | 7.5637 | 10.6151 | 9.1609 | 9.2107 |
| *BTun2* | 3.4557 | 12.9142 | 31.8984 | 15.746 | 12.9094 | 7.3399 |
| *BTgli* | 11.2748 | 5.3367 | 10.7084 | 6.8097 | 1.2769 | 0.8552 |
| *BTun1* | 0.0001 | 0.0001 | 0.0001 | 0.0001 | 0.0001 | 0.0001 |
| *BTace1* | 0.0001 | 0.0001 | 0.0001 | 0.0001 | 0.0001 | 0.0001 |
| *BTace3* | 1.4235 | 1.6692 | 0.9983 | 3.0894 | 2.8414 | 0.9596 |
| *BTace2* | 0.5058 | 0.6302 | 0.9039 | 1.7016 | 0.8029 | 0.5031 |
| *BTace4* | 0.6632 | 0.8838 | 0.9825 | 1.2813 | 0.7297 | 0.397 |
| *BTnrl9* | 3.3721 | 0.5935 | 3.0303 | 2.693 | 0.0001 | 0.0001 |
| *BTnrl2* | 5.6378 | 2.6416 | 5.1377 | 3.3826 | 0.4551 | 0.4069 |
| *BTnrl4* | 2.4948 | 1.1808 | 3.2793 | 1.9992 | 0.5664 | 0.6549 |
| *BTnrl3* | 3.823 | 1.4076 | 3.7602 | 2.7211 | 0.8321 | 0.6291 |
| *BTnrl10* | 7.7854 | 1.541 | 6.0723 | 4.8815 | 0.987 | 2.0774 |
| *BTnrl5* | 4.8387 | 1.4322 | 3.8212 | 3.41 | 1.1532 | 0.9427 |
| *BTnrl8* | 8.2445 | 1.5725 | 3.2117 | 4.275 | 2.0702 | 0.736 |
| *BTnrl7* | 2.8031 | 0.2894 | 3.6937 | 3.0783 | 0.4368 | 0.3177 |
| *BTnrl6* | 1.9952 | 1.1223 | 0.7019 | 2.19 | 0.4385 | 0.5839 |
| *BTnrl1* | 1.7745 | 0.4974 | 1.5951 | 0.7881 | 0.5564 | 0.5531 |

^1^E, Egg; N1-2, 1^st^- and 2^nd^-instar nymphs; N3, 3^rd^-instar nymph; N4, 4^th^-instar nymph; M, Male; F, Female.

**Table S4.** FPKM values from transcriptome data of COE genes in *B. tabaci* MED adults treated with an imidacloprid gradient.

| **Gene** | **IM0**^1^ | **IM25**^1^ | **IM50**^1^ | **IM100**^1^ |
| --- | --- | --- | --- | --- |
| *BTbe7* | 24.0534 | 29.0159 | 33.1026 | 33.0442 |
| *BTbe6* | 81.4236 | 131.0304 | 88.6651 | 176.5318 |
| *BTbe5* | 225.5412 | 285.2309 | 293.5594 | 319.0075 |
| *BTbe8* | 0.4995 | 0.4995 | 0.444 | 0.9435 |
| *BTbe2* | 3.848 | 6.375 | 4.3649 | 4.8818 |
| *BTbe4* | 81.4236 | 131.0304 | 88.6651 | 176.5318 |
| *BTbe3* | 22.3481 | 23.7199 | 26.7491 | 50.4689 |
| *BTbe1* | 18.5662 | 24.3042 | 22.5133 | 21.3804 |
| *BTbe9* | 17.4104 | 20.2754 | 17.9246 | 38.1265 |
| *BTbe10* | 10.3765 | 9.9179 | 8.2553 | 11.1791 |
| *BTbe13* | 5.4937 | 9.4422 | 6.4665 | 17.5109 |
| *BTbe11* | 25.4079 | 37.8231 | 30.6627 | 61.152 |
| *BTbe12* | 0.191 | 0.191 | 0.0001 | 0.2865 |
| *BTbe15* | 0.0001 | 0.0001 | 0.0525 | 0.0001 |
| *BTbe14* | 0.2894 | 0.6365 | 0.4629 | 0.2315 |
| *BTjhe1* | 133.4137 | 145.7553 | 172.2312 | 186.119 |
| *BTjhe2* | 24.0534 | 29.0159 | 33.1026 | 38.0442 |
| *BTjhe3* | 0.6294 | 0.4196 | 0.6294 | 0.5036 |
| *BTglt* | 0.0001 | 0.0001 | 0.0525 | 0.0001 |
| *BTae1* | 3.2916 | 3.9268 | 3.5803 | 5.3704 |
| *BTae2* | 4.2732 | 5.8323 | 6.0633 | 9.297 |
| *BTae3* | 0.2792 | 0.4653 | 0.9306 | 0.4653 |
| *BTae6* | 29.1768 | 33.8732 | 35.2822 | 29.7051 |
| *BTae4* | 1.9272 | 2.324 | 2.5507 | 2.2673 |
| *BTae5* | 1.9434 | 2.11 | 1.8324 | 2.2211 |
| *BTun2* | 45.9622 | 54.9087 | 61.7303 | 65.5325 |
| *BTgli* | 1.3058 | 1.4822 | 1.4822 | 1.4469 |
| *BTun1* | 1.4018 | 1.9829 | 2.359 | 1.5385 |
| *BTace1* | 0.0001 | 0.0001 | 0.0001 | 0.0001 |
| *BTace3* | 0.2615 | 0.0001 | 0.0437 | 0.0001 |
| *BTace2* | 0.3051 | 0.0437 | 0.0872 | 0.0437 |
| *BTace4* | 0.2615 | 0.0001 | 0.0437 | 0.0001 |
| *BTnrl9* | 1.6852 | 2.3079 | 2.8207 | 2.7475 |
| *BTnrl2* | 2.1566 | 2.7056 | 2.5879 | 2.9016 |
| *BTnrl4* | 4.8646 | 5.7892 | 6.5129 | 4.945 |
| *BTnrl3* | 0.2054 | 0.7188 | 0.2739 | 0.5135 |
| *BTnrl10* | 0.2464 | 0.7587 | 0.2821 | 0.5378 |
| *BTnrl5* | 3.1843 | 5.5415 | 6.2032 | 5.0866 |
| *BTnrl8* | 0.9791 | 1.0183 | 0.7442 | 1.2533 |
| *BTnrl7* | 2.4727 | 3.0772 | 3.0772 | 3.6816 |
| *BTnrl6* | 3.2603 | 4.0296 | 5.1285 | 5.0152 |
| *BTnrl1* | 12.1435 | 19.9797 | 12.403 | 28.698 |

^1^IM0, whitefly treated with water; IM25, whitefly treated with 25 mg/L imidacloprid; IM50, whitefly treated with 50 mg/L imidacloprid; IM100, whitefly treated with 100 mg/L imidacloprid.
